# Chrysin reduces inflammation and oxidative stress and improves ovarian function in D-gal-induced premature ovarian failure

**DOI:** 10.1080/21655979.2021.2005991

**Published:** 2022-03-24

**Authors:** Xinning Li, Xuelan Li, Ling Deng

**Affiliations:** Department of Reproductive Medicine Center, Shunde Hospital, Southern Medical University (The First People’s Hospital of Shunde), Foshan, Guangdong, China

**Keywords:** Chrysin, premature ovarian failure, inflammation, oxidative stress

## Abstract

Premature ovarian failure (POF), a frequently occurring pathology. Chrysin has antioxidant, anti-inflammatory, anti-apoptotic and other pharmacological activities. This study was designed to detect the effect of Chrysin on POF. The establishment of POF was depended on the subcutaneous injection of D-gal (200 mg/kg/d). With the adoption of ELISA, the levels of hormones and release of inflammatory cytokines were assayed. The expression of MDA, GSH-px, SOD and ROS was evaluated with corresponding kits. In addition, the pathological changes of ovary and apoptosis of ovarian granulosa cells in D-gal-induced mice were detected using H&E staining and TUNEL, respectively. Moreover, the levels of FSH receptor and apoptosis-related proteins were measured with western blot. Finally, ERβ expression was measured with RT-qPCR and western blot. In this study, we found that chrysin regulated the expression of hormones and weight of D-gal-induced mice. It was also found that chrysin inhibited the inflammation and oxidative stress in mice with D-gal induction. In addition, the number and advancement of follicle in D-gal-induced mice treated with chrysin revealed that chrysin could improve the ovarian function of mice with POF. Furthermore, chrysin exhibited inhibitory effects on the apoptosis of ovarian granulosa cells in D-gal-induced mice. More importantly, chrysin molecule targeted ERβ and activated ERβ expression in POF. Overall, Chrysin reduces inflammation and oxidative stress and improves ovarian function in D-gal-induced premature ovarian failure, suggesting that chrysin is valuable for the treatment of POF.

## Introduction

Premature ovarian failure (POF), also called primary ovarian insufficiency (POI) or premature menopause, is a gynecological disease contributing to amenorrhea, infertility, menopause as well as urogenital symptoms [1,2]. The typical feature of POF is that ovarian follicles quickly reduce with none or few residual follicles in women before or at the age of 40 [3]. It is estimated that the incidence of POF is as high as 1 in 100 when women aged 40 and by the age of 30, the incidence rate is as high as one in 1000 [4,5]. As survival rates for young cancer patients continue to increase, the strategy of management has been changed from cure with any cost to improving the quality of life [6]. However, the pathogenesis underlying POF remains largely unclear. Hormone replacement therapy (HRT) is available to treat the symptoms of POF, and follicle donation is available for several POF patients seeking to become pregnant [7]. However, HRT has been confirmed to confer a high risk of coronary heart disease, endometrial cancer and breast cancer in women with POF [8].

POF is a common clinical feature of galactosemia, and women with galactosemia eventually develop POF [9]. The estrous cycle of female mice is similar to but shorter than that of humans. D-galactose (D-gal)-induced POF model is used as a model of aging in mice and has been widely used to study the mechanisms underlying ovarian aging, due to the accelerated aging observed in this model is very similar to observations in humans [10]. Many studies have indicated that advanced glycation end products (AGEs) and reactive oxygen species (ROS) exacerbate and accelerate the aging process and contribute to the early phases of age-related diseases [11]. D-gal administration can cause excessive ROS formation and AGE accumulation, which are widely accepted as causes of aging that gradually damage ovarian functions [12].

Chrysin (5, 7-dihydroxyflavone) is a flavonoid extracted from honey, propolis, and passion flowers [13]. Chrysin is used to treat liver diseases, neurodegenerative diseases, and reproductive system diseases [14]. Recent studies have revealed the numerous biological properties of chrysin including antioxidant, anti-apoptosis, anti-inflammation, and anti-cancer [15,16]. Moreover, the protective effects of chrysin agianst oxidative stress in D-galactose-induced aging rats have been demonstrated. The data showed that co-treatment with D-gal and chrysin by oral gavage for 8 weeks increased antioxidant enzyme activities and decreases MDA levels [17]. However, the effect and mechanism of chrysin on inflammatory and oxidative stress by which D-galactose alters premature ovarian failure remains unknown.

Consequently, the aim of this study was to explore the temporal pattern of D-gal treatment and to investigate whether chrysin administration could be effective in the preservation of the ovarian function damaged by D-gal treatment, elucidating the underlying molecular mechanisms focusing on inflammatory, apoptotic, and TGF-β signaling pathways.

## Material and methods

### Preparation of POF mice model

One hundred C57BL/6 female mice aged 7–8 weeks that procured from the Animal Core Facility were housed under a temperature- and humidity-controlled environment (a 12-h/12-h light/dark cycle) with abundant supply of water and food.

One hundred mice were randomly separated into five groups and each group had 20 mice. The POF mice model was established by subcutaneous injection of D-galactose (D-gal, Sigma Aldrich, Shanghai, China) with a dose of 200 mg/kg/d for 42 days. In different D-gal + Chrysin groups, mice were administrated with different doses of chrysin (Sigma Aldrich, Shanghai, China, 36.1 mg/kg/day, 72.2 mg/kg/day, 144.4 mg/kg/day) [18]. All animal procedures were operated according to the NIH Guide for the Care and Use of Laboratory Animals approved by the ethical guidelines of Shunde Hospital, Southern Medical University (The First People’s Hospital of Shunde).

### Evaluation of relative organ weights

On the last day of drug administration, all mice were sacrificed with anesthesia. Thereafter, the connective tissues of uteri and ovaries were removed and weighed. The relative weight of each organ was calculated relative to the final weight before euthanasia and displayed as mg/100 g body weight.

### Follicle counts

In all ovarian sections, the fifth incision with a thickness of 4 μm was selected to calculate the number of follicles in each follicle category and the follicular development of 6 sections was evaluated with the application of a digital camera installed on an optical microscope [19].

### Enzyme-Linked Immunosorbent Assay (ELISA)

With the application of ELISA kits (Beyotime, Haimen, China), the levels of hormones, including 17β-estradiol (E2), follicle-stimulating hormone (FSH), progesterone (P) as well as anti-Mullerian hormone (AMH), and the releases of inflammatory cytokines, such as TNF-α (tumor necrosis factor-α), IL-1β (Interleukin-1β) and IL-6 (Interleukin-6) were detected strictly in line with the manufacturer's specification. Finally, the optical density at 450 nm was recorded with the help of Varioskan LUX (Invitrogen; Thermo Fisher Scientific).

### Hematoxylin and Eosin (H&E) staining

Ovaries of each mouse got fixed with 4% formaldehyde, dehydrated in ethanol, embedded with paraffin, and sliced into 4 µm sections. Subsequently, the sections were stained with the adoption of commercial H&E kits (Solarbio, Beijing, China). Under an Olympus CX21 microscope (Olympus, Japan), the images of ovaries were visualized [20].

### Terminal-deoxynucleoitidyl Transferase Mediated Nick End Labeling (TUNEL)

The apoptosis of ovarian granulosa cells in D-gal-induced mice was assessed by TUNEL (Beyotime, Shanghai, China) [21]. In brief, the fixation and permeability of ovarian granulosa cells were conducted with 4% paraformaldehyde (Sigma-Aldrich) and 0.25% Triton‐X 100 (Sigma-Aldrich), respectively. Then, the cells were washed with phosphate buffered saline (PBS), after which DAPI was used to counterstain the nucleus for 10 min. Finally, the positive apoptotic cells were observed under a florescent microscope (Olympus, Tokyo, Japan).

### Western blot analysis

Total proteins were isolated with radioimmunoprecipitation assay lysing solution (Thermo Fisher Scientific, Inc.) and quantified with Bicinchoninic acid (Pierce, USA) methods. Separated by 12% sodium dodecyl sulfate/polyacrylamide gel electrophoresis (SDS-PAGE), the proteins were then transferred onto polyvinylidene difluoride (PVDF) membranes (Roche, Basel, Switzerland). After impeded with 5% nonfat milk, the membranes were incubated with primary antibodies against Bax (1:1000, No. ab182733, Abcam, Cambridge, UK), Bcl-2 (1:1000, No. 15,071, Cell Signaling Technology, Inc., Danvers, MA, USA), cleaved caspase-3 (1:400; No. ab32042, Abcam, Cambridge, UK) and FSH receptor (1:500, No. ab75200, Abcam, Cambridge, UK) at 4°C overnight. GAPDH (1:1000, No. ab181602, Abcam, Cambridge, UK) was considered as an internal reference control. On the next day, the membranes were incubated with a horseradish peroxidase-conjugated rabbit anti-rat IgG secondary antibody (1:1000; No. ab6734, Abcam, Cambridge, UK). At last, the protein bands were visualized with the application of an enhanced chemiluminescence system (Beyotime, Shanghai, China).

### Reverse transcription-quantitative PCR (RT-qPCR)

Total RNA was extracted with Trlzol® reagent (Thermo Fisher Scientific, Inc.) and reversely transcribed into complementary DNA (cDNA) by PrimerScript reverse transcriptase (Takara, Tokyo, Japan). Subsequently, SYBR Premix Ex Taq reagent (Takara, Tokyo, Japan) was applied to perform qPCR reaction on an ABI 7500 quantitative PCR instrument (ABI/Perkin Elmer, CA, USA). At last, the relative gene expression was determined with the adoption of 2^−ΔΔCt^ methods [22].

### Reactive oxygen species (ROS) measurement

The content of reactive oxygen species (ROS) in cell suspension was assayed by ROS kits (Shanghai Yanjin Biological Technology Co., Ltd., Shanghai, China). 2′,7′-dichlorofluorescin diacetate (DCFH-DA) was utilized to stain the cells for 30 min. Finally, the content of ROS was evaluated with the application of flow cytometry BD Biosciences, USA) when re-suspension in PBS was completed.

### MDA, SOD and GSH-px measurement

The expression of malondialdehyde (MDA), superoxide dismutase (SOD) as well as glutathione peroxidase (GSH-Px) in cell suspension were determined using MDA kits, SOD kits and GSH-Px kits (Beyotime, Shanghai, China), respectively.

### Molecular docking

The structure of chrysin was drawn in the ChemDraw software, and then imported into OpenBabel software (v2.2.1) for hydrogenation and converted into a mol2 format file. Subsequently, the structure of ERβ (PDB: 2I0G) was obtained from the RCSB PDB webpage (https://www.rcsb.org/). Thereafter, the protein ERβ file was opened in PyMOL software (v2.2.0) to remove the excess water molecules, delete any irrelevant small ligands originally carried, and only keep the protein structure. Because the downloaded protein structure has ligands, the original ligands are deleted and the original ligand positions are set as docking sites. AutoDock (v4.2) was utilized to display the specific docking energy value after running. Finally, the results were analyzed with the adoption of Protein-Ligand Interaction Profiler (PLIP; https://plip-tool.biotec.tu-dresden.de/plip-web).

### Statistical analysis

All data collected from experiments were presented as the mean ± standard deviation (SD) and analyzed with SPSS 20.0 (SPSS Inc., Chicago, USA). Student’s t test was used for comparisons between two groups and one-way analysis of variance (ANOVA) was applied for multiple groups. P less than 0.05 was viewed to be statistically important.

## Results

### Chrysin regulated the body weight of D-gal-induced mice

**Figure 1.**
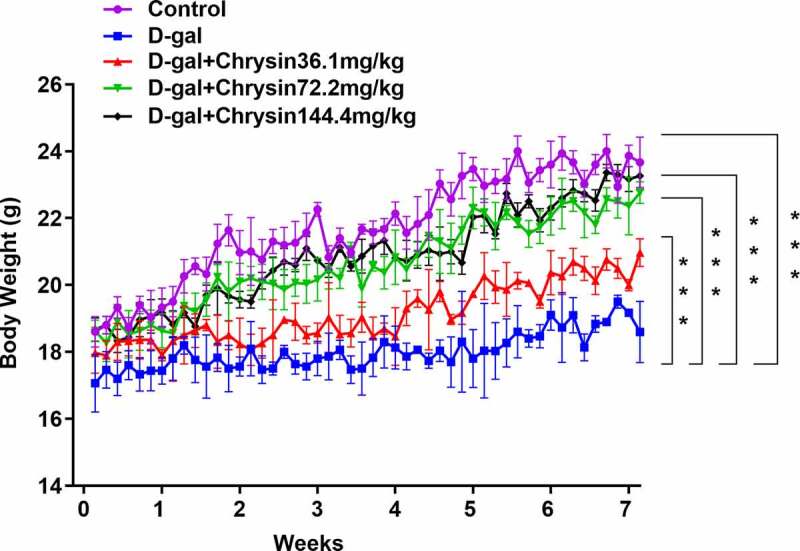
**Chrysin regulated the body weight of D-gal-induced mice**. The weight of D-gal-induced mice. ***P < 0.001.

As Figure 1 depicts, in the D-gal group, there was an obvious weight loss compared with control group. Chrysin treatment significantly and concentration-dependently increased the mice weight compared to the D-gal group.

### Chrysin regulated the levels of hormones in D-gal-induced mice

**Figure 2.**
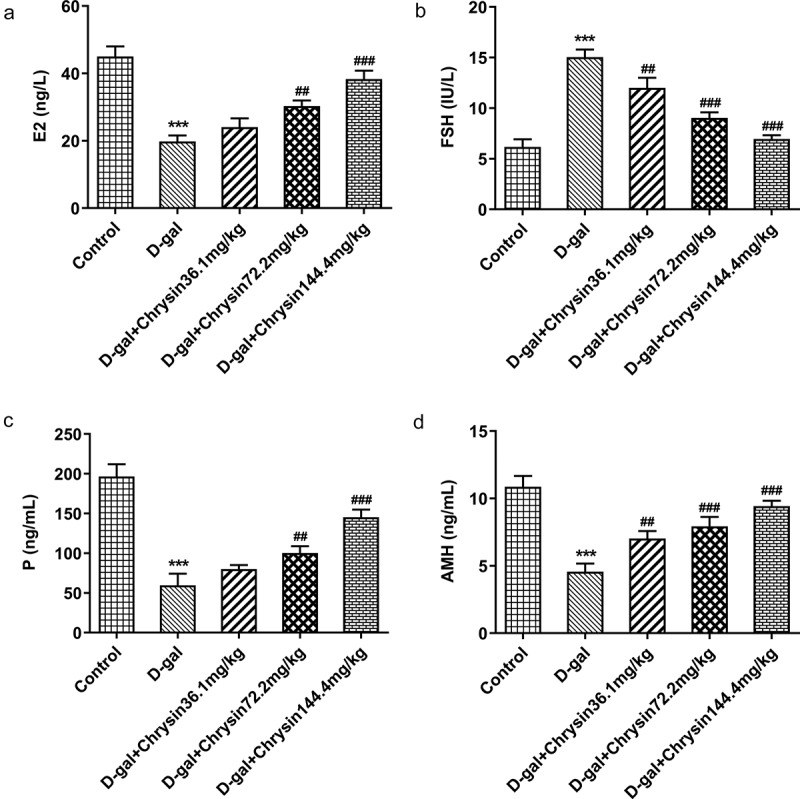
**Chrysin regulated the levels of hormones in D-gal-induced mice**. (a-d) The levels of E2, FSH, P and AMH were detected using ELISA. ***P < 0.001 vs Control; ^##^P < 0.01, ^###^P < 0.001 vs D-gal.

With the application of ELISA, the levels of E2, FSH, P and AMH were evaluated. The D-gal treatment group had significantly increased serum FSH level and drastically decreased E2, P and AMH levels compared to those in the control (Figure 2a-d). Compared with the POF model group, chrysin treatment markedly and concentration-dependently decreased the serum FSH level and increased the serum E2, P and AMH levels.

### Chrysin inhibited the inflammation and oxidative stress in D-gal-induced mice

**Figure 3.**
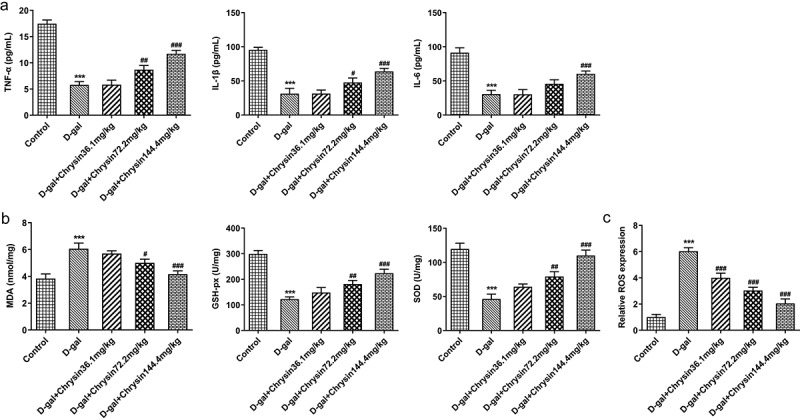
**Chrysin inhibited the inflammation and oxidative stress in D-gal-induced mice**. The expression of TNF-a, IL-1β and IL-6 was measured using ELISA. (b) The expression of MDA, GSH-px and SOD was detected using corresponding kits. (c) The expression of ROS was detected by DCH-DA fluorescence staining. ***P < 0.001 vs Control; ^#^P < 0.05, ^##^P < 0.01, ^###^P < 0.001 vs D-gal.

As a key inflammatory cytokine the levels of TNF-α, IL-1β and IL-6 in ovarian tissues were assessed using ELISA. Compared with control group, the levels of TNF-α, IL-6 and IL-1β were significantly elevated in the ovaries of mice treated with D-gal. Conversely, chrysin treatment significantly inhibited the elevation of TNF-α, IL-1β and IL-6 levels in D-gal group and reversed them close to that of controls, D-gal+Chrysin144.4 group was closer to controls compared with D-gal+Chrysin36.1 and D-gal+Chrysin72.2 groups (Figure 3(a)).

To further investigate the effects of chrysin on oxidative stress, the activities of MDA, GSH-px and SOD in D-gal-induced mice were assessed with corresponding kits. Compared with Control, D-gal upregulated MDA expression but downregulated GSH-px and SOD expression (Figure 3(b)). However, chrysin treatment reversed the effects of D-gal induction on MDA, GSH-px and SOD, evidenced by the downregulated MDA expression and upregulated GSH-px and SOD expression. In addition, we observed an increase in ROS production after D-gal stimulation, which was obviously and concentration-dependently reversed by chrysin treatment (Figure 3(c)).

### Chrysin improved follicular development and increased follicular number in D-gal-induced mice

**Figure 4.**
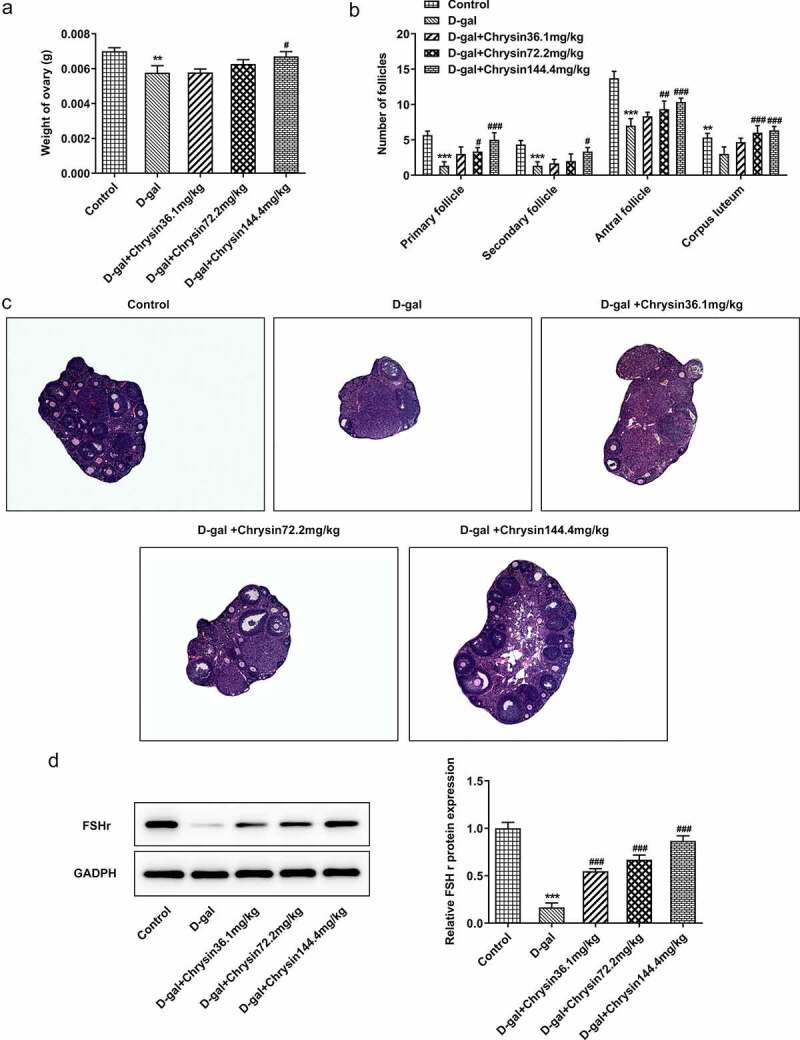
**Chrysin improved follicular development and increased follicular number in D-gal-induced mice**. (a) The ovarian weight of D-gal-induced mice. (b) The number of follicles in D-gal-induced mice. (c) Pathological changes of ovaries in mice were detected by HE staining. D. Western blot was used to detect the expression of FSH receptor. **P < 0.01, ***P < 0.001 vs Control; ^#^P < 0.05, ^##^P < 0.01, ^###^P < 0.001 vs D-gal.

Female mice treated with D-gal showed significant reduction in ovaries. On the other hand, chrysin treatment significantly counteracted these effects and maintained ovarian weights (Figure 4(a)). It was noted that the effects of chrysin on ovaries weight were in a concentration-dependent manner. The classification of follicle was based on the characteristics proposed by Hirshfield & Midgley [1978, 23]. According to Figure 4(b), counting of the primordial follicles showed that D-gal treatment reduced the proportion of primordial follicles compared to that in the control group. Significantly more follicles were counted at the different developmental stages of maturation in the chrysin treatment group than in the D-gal model group.

In Figure 4(c), the control group and the D-gal+ chrysin group showed normal ovarian morphological structure with different stages of follicular development. However, in the D-gal group, almost all follicles were atresia, and no primordial follicles were observed. In addition, it was found that the granular layer in the atresia follicle was generally loose. The growing follicles have a relatively irregular arrangement with discrete membrane layers, and the thickness of zona pellucida is uneven in some follicles. It was found that most primary follicles lacked oocytes and the interstitial structure of ovary was destroyed. In addition, collagen fiber accumulation in the form of vascular congestion, hemorrhage and fibrosis were detected in the matrix.

According to western blot, the results indicated that the expression of follicle-stimulating hormone receptor (FSHR) in D-gal group was extensively dropped off in comparison with the control. As revealed in Figure 4(d), FSHR was significantly and concentration-dependently risen compared with D-gal group after chrysin treatment.

### Chrysin inhibited the apoptosis of ovarian granulosa cells in D-gal-induced mice

**Figure 5.**
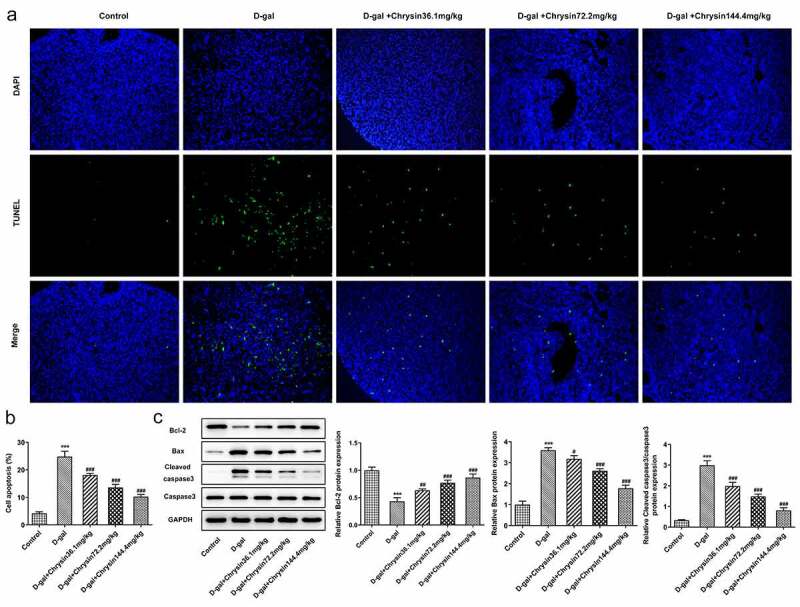
**Chrysin inhibited the apoptosis of ovarian granulosa cells in D-gal-induced mice**. (a-b) TUNEL was used to detect the cell apoptosis. (c-d) The levels of apoptosis-related proteins were detected using western blot. ***P < 0.001 vs Control; ^#^P < 0.05, ^##^P < 0.01, ^###^P < 0.001 vs D-gal.

With the application of TUNEL assay, the apoptosis of ovarian granulosa cells was detected. As Figure 5(a-B) displayed, D-gal induction greatly enhanced the number of TUNEL-positive cells compared with that in Control group. However, the increased cell apoptosis was then significantly decreased by chrysin, suggesting that chrysin exerted protective effects against D-gal-induced ovarian cell apoptosis. Compared with the Control, D-gal induction downregulated Bcl2 expression but upregulated the expression of Bax and cleaved caspase3, which were then reversed by chrysin (Figure 5(c-D)). Notably, chrysin inhibited the apoptosis of ovarian granulosa cells in POF.

### Chrysin molecule targeted ERβ and activated ERβ expression in POF

**Figure 6.**
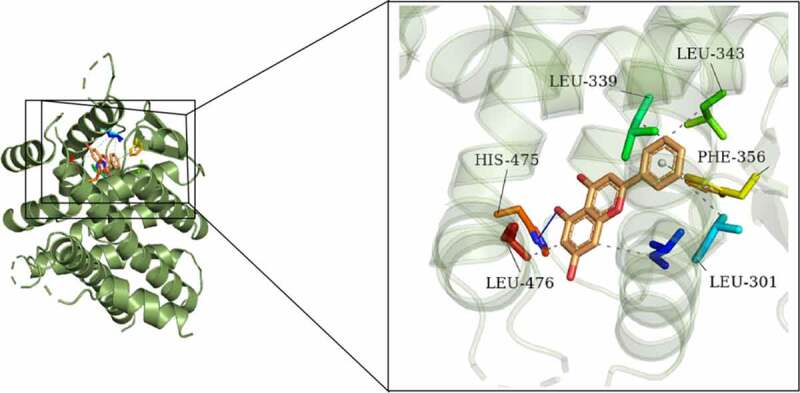
**Molecular docking diagram**. Chrysin was verified to be docked with ERβ. In the activate site of LEU-339, LEU-343, HIS-475, PHE-356, LEU-476 and LEU-301 bonds with chrysin.

**Figure 7.**
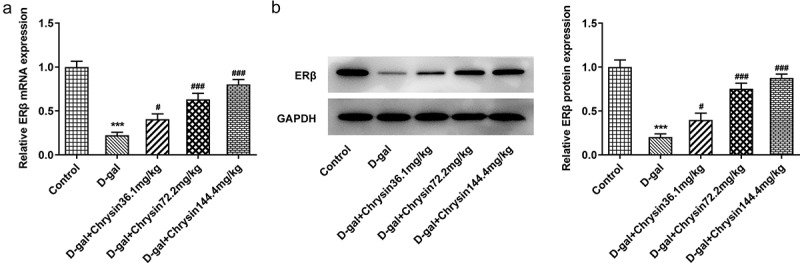
**Chrysin molecule targeted ERβ and activated ERβ expression in premature ovarian failure**. The mRNA (a) and protein (b) levels of ERβ were detected using RT-qPCR and western blot, respectively.

One previous study has found that genistein can activate the expression of ERβ when improving radiation-induced POF, which indicates that ERβ activation plays a vital role in POF [24]. Besides, according to Targetnet database (http://targetnet.scbdd.com/), chrysin could target ERβ (Figure 6). In view of this, we also investigated the relationship between chrysin and ERβ in POF. As shown in Figure 7(a), chrysin could be docked with ERβ, and the docking result is −8.3. In addition, the decreased ERβ expression in D-gal-induced mice was then increased by chrysin administration (Figure 7(b)).

## Discussion

As a rare heterogeneous disorder, POF is featured by cessation of menstruation occurring in women younger than 40 years old [25]. In 1% POF women, POF occurs due to autoimmune ovarian damage or genetic aberrations involving the X chromosome, autosomes, or some specific genes [26]. What’s worse, women with POF are often neglected and under-treated, which represents many clinical management challenges [27,28]. Currently, the effective methods for the treatment of POF haven’t shown up. Considering this, we conducted this study by establishing D-gal-induced mice model to simulate POF in vitro. In this study, we found that D-gal regulated hormone levels, promoted inflammation, oxidative stress and granulosa cell apoptosis as well as damaged follicular development.

Chrysin, a member of flavonoids, can be extracted from many plants and protect against inflammatory response, tumor and oxidative stress [29,30]. In the present study, it was found that chrysin exhibited inhibitory effects on inflammation and oxidative stress in D-gal-induced mice. Besides, chrysin has been verified to exert protective effects on radiation-induced ovarian damage [18]. La Marca A holds the opinion that galactose toxicity attenuates FSH bioactivity and inhibits E2 production from granulose cells [31]. As a significant early marker of ovarian aging, AMH reflects the size of the ovarian follicle pool [32]. In the present study, we found that chrysin significantly increased ovarian AMH expression as well as the number of primordial, primary and secondary follicles, implying that chrysin promoted the development of follicular and the maintenance of primordial follicles.

Previous studies have reported that oxidative stress can reduce the number of follicles and oocytes [33,34]. In the present study, we found that chrysin enhanced the activities of SOD and GSH-px but reduced MDA level. In D-gal-induced mice model, chrysin exerted protective effects by inhibiting oxidative stress and its effects might be mediated via the suppression of ROS. Additionally, inflammatory response acts as a significant player in ovarian follicular loss [18]. An increasing number of reports have evidenced that aberrant inflammation can alter normal ovarian follicular dynamics contributing to infertility [35,36]. In our study, chrysin treatment significantly and concentration-dependently inhibited the elevation of TNF-α, IL-1β and IL-6 levels in D-gal-induced mice, indicating that chrysin relieved potent expression of the inflammatory markers in the ovaries of D-gal induced mice.

As reported, the follicles become atretic when 10% of granulosa cells have undergone apoptosis [37]. The importance of granulosa cell apoptosis in the advancement of follicular atresia has been also confirmed by Chu YL, et al [38]. Our study demonstrated that D-gal induction increased the number of apoptotic granulosa cells and atretic follicles, which was subsequently diminished by chrysin treatment. Besides, the downregulated Bcl-2 and upregulated Bax and cleaved caspase-3 expression caused by D-gal induction were reversed after chrysin treatment, indicating that chrysin attenuated D-gal-induced POF by activating cell apoptosis.

It has been found that genistein can activate ERβ expression when improving radiation-induced POF, indicating that activation of ERβ plays a role in POF [24]. We predicted that Chrysin targeted the expression of ERβ through the TargetNet database (http://targetnet.scbdd.com). Therefore, in this paper, molecular docking was used to demonstrate the relevant targeting studies of Chrysin and ERβ. We found that Chrysin molecule targets ERβ, and we found a significant decrease of ERβ expression in D-gal induced, while Chrysin reversed ERβ expression. Therefore, we preliminarily concluded that Chrysin molecules target ERβ and activate ERβ expression in POF.

## Conclusion

To sum up, chrysin effectively inhibited D-gal-induced oxidative stress, inflammatory response, apoptosis as well as ovarian injury, implying that chrysin might serve as a potential protective agent against POF.

## Data Availability

The datasets analyzed during the current study are available from the corresponding author on reasonable request.

## Research highlights

Chrysin molecule targetes ERβ and activated ERβ expression in POF

Chrysin reduces inflammation and oxidative stress in D-gal-induced POF

Chrysin improves ovarian function in D-gal-induced POF

## Declarations

All authors agree on the data and publication of the article

## Availability of data and materials

The analyzed data sets generated during the present study are available from the corresponding author on reasonable request.
